# Matrix Metalloproteinase-3 -1171 5A/6A Polymorphism (rs35068180) is Associated with Risk of Periodontitis

**DOI:** 10.1038/srep11667

**Published:** 2015-06-30

**Authors:** Cheng Ding, Xing Chen, Peng-tao Zhang, Jin-ping Huang, Yan Xu, Ning Chen, Liang-jun Zhong

**Affiliations:** 1 Department of Stomatology, The Affiliated Hospital of Hangzhou Normal University, Hangzhou, China; 2 Physical Examination Center, The Affiliated Hospital of Hangzhou Normal University, Hangzhou, China; 3 Jiangsu Key Laboratory of Oral Diseases, Nanjing Medical University, Nanjing, China; 4 Hangzhou Geriatric Hospital, Hangzhou, China; 5 School of Medicine, Hangzhou Normal University, Hangzhou, China

## Abstract

Matrix metalloproteinase-3 (MMP3) plays a key role in tissue degradation in periodontitis. The relationship between the *MMP3* -1171 5A/6A polymorphism (rs35068180) and periodontitis has been widely studied. However, existing studies have yielded contradictory results. We therefore conducted a meta-analysis to comprehensively investigate these inconclusive findings. Several electronic databases were searched for eligible articles. Seven case-control studies from 6 articles were searched without any language restrictions. Pooled estimates indicated that *MMP3* -1171 5A/6A polymorphism is associated with a decreased risk of periodontitis (allelic genetic model: OR = 0.70, 95% CI: 0.62–0.80, *P*_heterogeneity_ = 0.315; heterozygous model: OR = 0.50, 95% CI: 0.39–0.65, *P*_heterogeneity_ = 0.221; homozygous model: OR = 0.42, 95% CI: 0.25–0.69, *P*_heterogeneity_ = 0.265; dominant model: OR = 0.49, 95% CI: 0.38–0.62, *P*_heterogeneity_ = 0.238, respectively). Similar results were also found in chronic periodontitis (CP), Asian, Asian&CP, and non-smokers subgroups. Moreover, *MMP3* rs35068180 polymorphism might be associated with a lower risk of aggressive periodontitis (AgP) in Asians (allelic genetic model: OR = 0.66, 95% CI: 0.48–0.91, *P*_heterogeneity_ = 0.945), and CP in Caucasians and Brazilians. In conclusion, this meta-analysis demonstrates that *MMP3* -1171 5A/6A polymorphism may be associated with decreased risk of both CP and AgP in Asians. Large independent studies to replicate these results are necessary to validate these associations in other populations.

Periodontitis is a globally prevalent inflammatory disease that affects the tooth-supporting apparatus^1^. Periodontitis is extremely common; chronic periodontitis (CP) is the most common form of the disease and affects between 5.0% and 79.6% of the world’s population^2^. CP is also the major cause of tooth loss, particularly in the elderly. Aggressive periodontitis (AgP), which generally presents in early adulthood, is characterised by a rapid, severe destruction of alveolar bone and periodontal attachment loss^3^. Although microorganisms are thought to be the prerequisite factor^4^, environmental (e.g., stress and smoking)^5^,^6^ and genetic factors (e.g., *IL-1*, *IL-8* and *TGF-β1*)^7^,^8^,^9^ are also involved in the pathogenesis of periodontitis.

Matrix metalloproteinases (MMPs) are the most important pathway in periodontitis-associated tissue breakdown, owing to their role in the pathological destruction of extracellular matrix and immune responses related to periodontal inflammation^10^. MMP3 (stromelysin-1) can degrade collagen in the basal membrane and can induce the synthesis of other MMPs, such as MMP1 and MMP9. The *MMP3* gene, located on chromosome 11q22.2–22.3 (adjacent to the *MMP1* and *MMP12* genes), is an endopeptidase produced by smooth muscle cells, macrophages and synovial cells^11^. The insertion/deletion of a single adenosine (5A/6A) at position -1171 of the *MMP3* promoter region (rs35068180 or rs3025058) can alter its transcription levels^12^. This may affect its ability to degrade connective tissue and lead to the progression of periodontitis. A prioritisation analysis of candidate genes for periodontitis using multiple computational tools identified *MMP3* as one of the most promising genes to be involved in periodontitis^13^.

Over the past decade, many genetic association studies have been conducted to evaluate the association between *MMP3* -1171 5A/6A polymorphism and the risk of periodontitis. However, the results of these studies were inconsistent. The discrepancies among studies may be due to the relatively small sample size in each investigation, as well as varying population characteristics. Therefore, we performed a meta-analysis to investigate the potential association of *MMP3* -1171 5A/6A polymorphism with the risk of periodontitis.

## Results

### Characteristics of eligible studies

**Figure 1:**
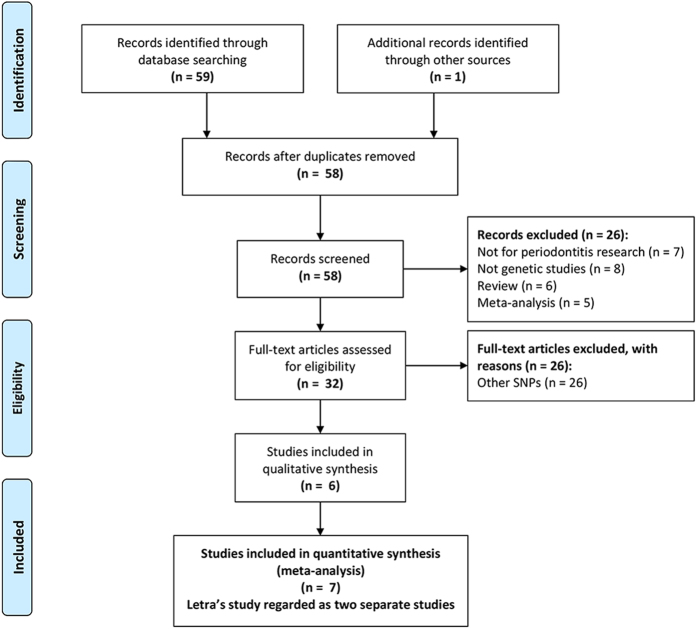
PRISMA flowchart for inclusion of studies in the meta-analysis.

Seven case-control studies from six publications were identified in the meta-analysis (a flow diagram is shown in Fig. 1). Basic characteristics and the quality assessment scores of the selected articles are all listed in Table 1. Overall, all of the included studies were moderate- to high-quality, with a mean quality score of 29.1/40 (range from 27 to 31). Of these case-control studies, four studies including 963 cases and 1227 controls were undertaken in Asians^14^,^15^,^16^,^17^, two studies containing 183 cases and 402 controls were conducted in a mixed Brazilian population^18^,^19^, and one study with 67 cases and 202 controls was performed in a Caucasian population^19^. Three studies enrolled non-smokers, which permitted smoking to be analysed as a risk factor^14^,^16^,^18^. Five eligible studies mentioned quality controls of the genetic analyses, such as blind genotyping, validation of genotyping accuracy, and random replications^15^,^16^,^17^,^19^. Two studies supplied only the allele frequency to compute OR in the allelic genetic model^15^,^19^. The control populations of three studies were not consistent with Hardy–Weinberg equilibrium (HWE)^16^,^17^,^18^. The minor allele frequency (MAF) for controls and genotype distributions of analysed polymorphism in different ethnicities are all listed in Table 2.

### Quantitative data synthesis

Between-study heterogeneity was not evident in all five comparisons (Table 3). Overall, we found that with the exception of a marginal association in the recessive model (6A/6A *vs*. 5A/5A + 5A/6A: OR = 0.74, 95% CI = 0.53–1.02, *P*_heterogeneity_ = 0.539, *p* = 0.06), the other four genetic models contributed to decreased susceptibility to periodontitis (6A allele *vs.* 5A allele: OR = 0.70, 95% CI = 0.62–0.80, *P*_heterogeneity_ = 0.315; 5A/6A *vs*. 5A/5A: OR = 0.50, 95% CI = 0.39–0.65, *P*_heterogeneity_ = 0.221; 6A/6A *vs*. 5A/5A: OR = 0.42, 95% CI = 0.25–0.69, *P*_heterogeneity_ = 0.265; 5A/6A + 6A/6A *vs*. 5A/5A: OR = 0.49, 95% CI = 0.38–0.62, *P*_heterogeneity_ = 0.238, respectively). After the HWE-violating studies were excluded, a significant association was found in the allele contrast (6A allele *vs.* 5A allele: OR = 0.80, 95% CI = 0.67–0.96, *P*_heterogeneity_ = 0.655).

**Figure 2:**
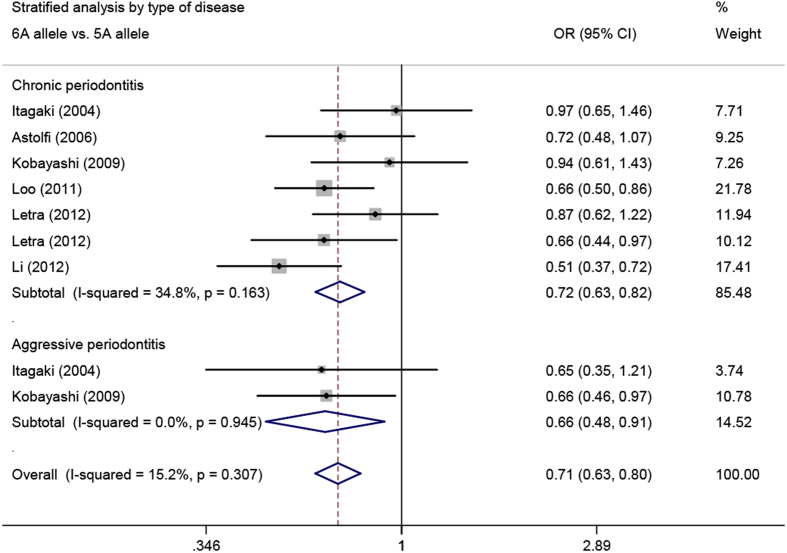
Meta-analysis of *MMP3* -1171 5A/6A polymorphism with chronic and aggressive periodontitis.

When stratified by the type of periodontitis, we found a significant association of the -1171 5A/6A polymorphism in the *MMP3* promoter region with both CP (6A allele *vs.* 5A allele: OR = 0.72, 95% CI = 0.63–0.82, *P*_heterogeneity_ = 0.163; 5A/6A *vs*. 5A/5A: OR = 0.50, 95% CI = 0.39–0.64, *P*_heterogeneity_ = 0.345; 6A/6A *vs*. 5A/5A: OR = 0.41, 95% CI = 0.25–0.68, *P*_heterogeneity_ = 0.336; 5A/6A + 6A/6A *vs*. 5A/5A: OR = 0.48, 95% CI = 0.38–0.62, *P*_heterogeneity_ = 0.317, respectively) and AgP (6A allele vs. 5A allele: OR = 0.66, 95% CI = 0.48–0.91, *P*_heterogeneity_ = 0.945) (Fig. 2).

**Figure 3:**
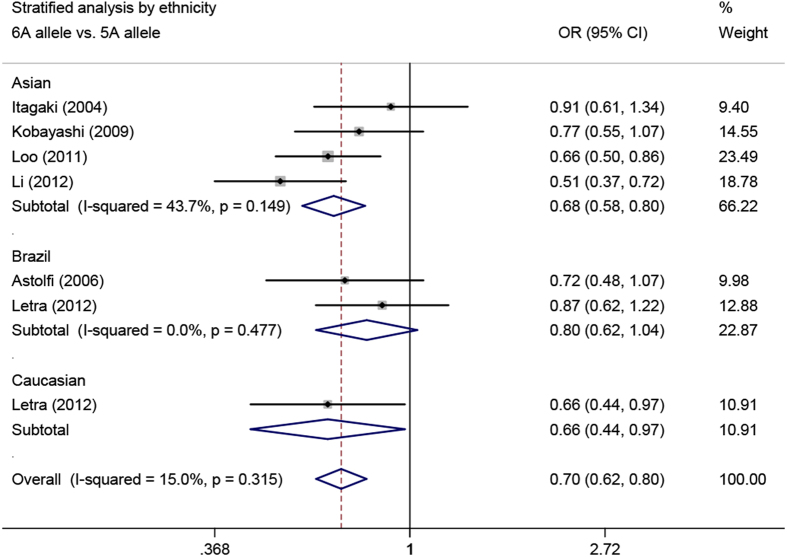
Meta-analysis of *MMP3* -1171 5A/6A polymorphism with periodontitis in different ethnicities.

In the ethnicity-stratified analysis, the associations were significant in Asians (6A allele *vs.* 5A allele: OR = 0.68, 95% CI = 0.58–0.80, *P*_heterogeneity_ = 0.149; 5A/6A *vs*. 5A/5A: OR = 0.52, 95% CI = 0.40–0.68, *P*_heterogeneity_ = 0.198; 6A/6A *vs*. 5A/5A: OR = 0.45, 95% CI = 0.25–0.81, *P*_heterogeneity_ = 0.173; 5A/6A + 6A/6A *vs*. 5A/5A: OR = 0.51, 95% CI = 0.39–0.66, *P*_heterogeneity_ = 0.202, respectively), Caucasians (all CP cases) (6A allele vs. 5A allele: OR = 0.66, 95% CI = 0.44–0.97), and Brazilian mixed populations (all CP cases) (5A/6A *vs*. 5A/5A: OR = 0.31, 95% CI = 0.13–0.77; 6A/6A *vs*. 5A/5A: OR = 0.32, 95% CI = 0.12–0.89; 5A/6A + 6A/6A *vs*. 5A/5A: OR = 0.31, 95% CI = 0.13–0.76, respectively) (Fig. 3). Furthermore, after excluding the 209 AgP cases in Asians, the magnitude of the effect on CP was similar in Asians (Table 3).

**Figure 4:**
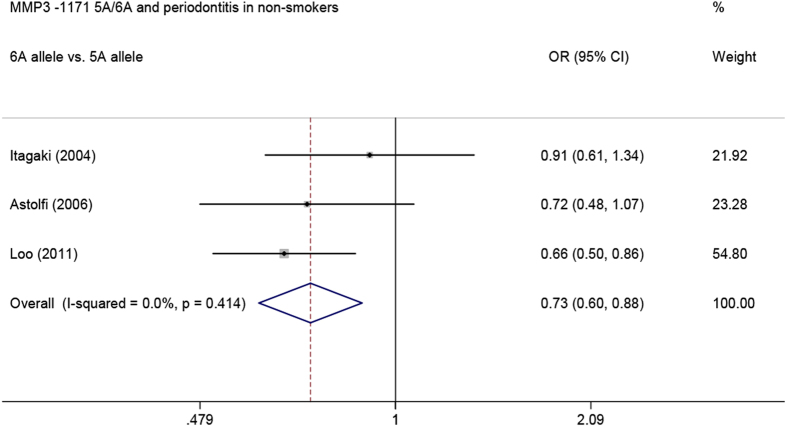
Meta-analysis of *MMP3* -1171 5A/6A polymorphism with periodontitis in nonsmokers.

In addition, when analysis was limited to the three studies that recruited non-smokers, we also found that the -1171 5A/6A polymorphism was associated with a decreased risk of periodontitis in nonsmokers (6A allele *vs.* 5A allele: OR = 0.73, 95% CI = 0.60–0.88, *P*_heterogeneity_ = 0.414; 5A/6A *vs*. 5A/5A: OR = 0.54, 95% CI = 0.39–0.75, *P*_heterogeneity_ = 0.151; 6A/6A *vs*. 5A/5A: OR = 0.50, 95% CI = 0.28–0.89, *P*_heterogeneity_ = 0.260; 5A/6A + 6A/6A *vs*. 5A/5A: OR = 0.53, 95% CI = 0.39–0.73, *P*_heterogeneity_ = 0.189, respectively) (Fig. 4).

### Sensitivity analysis and publication bias

Sensitivity analysis was performed by omitting individual studies to assess the effect of each publication on the overall results. No single study changed the pooled ORs qualitatively, which suggests that the results of our meta-analysis are accurate.

**Figure 5:**
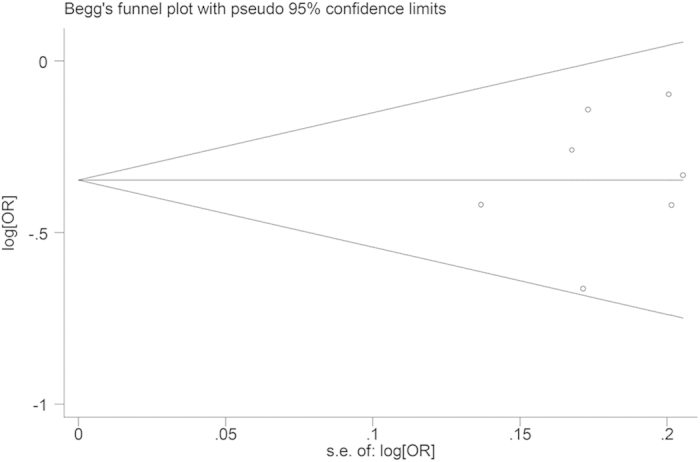
Funnel plot of *MMP3* -1171 5A/6A for the allelic model (6A allele *versus* 5A allele). SE = standard error.

Begger’s funnel plot and Egger’s test were performed to detect the publication bias of included studies. As shown in Fig. 5, the shape of the funnel plot did not reveal any evidence of obvious asymmetry. In addition, the results of Egger’s test did not show any evidence of publication bias.

## Discussion

Increasing evidence suggests that MMPs play a pivotal role in periodontitis^10^. Meta-analyses have demonstrated that both *MMP1* -1607 1G/2G and *MMP9* -1562C/T polymorphisms may be involved in the development of CP^20^,^21^. *MMP3* -1171 5A/6A and *MMP1* -1607 1G/2G (rs1799750) are in a strong linkage disequilibrium^22^. The relationship between *MMP3* -1171 5A/6A polymorphism and periodontitis has been widely investigated. However, previous studies have yielded contradictory results. We therefore performed this meta-analysis to comprehensively evaluate these inconclusive findings. To the best of our knowledge, this is the first meta-analysis to have investigated the association between *MMP3* -1171 5A/6A polymorphism and the susceptibility to periodontitis. Our meta-analysis was based on seven case–control studies that focused on the link between *MMP3* -1171 5A/6A polymorphism and periodontitis risk and included a total of 1,213 cases and 1,831 controls. The results of our meta-analysis revealed that -1171 5A/6A polymorphism is associated with a decreased risk of overall periodontitis; this association was consistently significant in all subgroups in analyses that were stratified by periodontitis type, ethnicity and smoking status.

Although phenotypic differences exist between AgP and CP in terms of the genetic predisposition to periodontitis, some scholars believe that both chronic and aggressive periodontitis may share some susceptibility genes^23^. Furthermore, some genetic polymorphisms (e.g., *TNF-α* -308G/A and *Fcγ* receptor IIIB NA1/NA2) have been shown to have similar effects on AgP and CP^24^,^25^. In this report, we first analysed the effect of *MMP3* -1171 5A/6A polymorphism on two forms of periodontitis. We found that this locus was associated with a decreased risk of periodontitis under four genetic models. Additionally, -1171 5A/6A showed a trend towards protective factor for periodontitis in the recessive model, although the finding was not statistically significant (*p* = 0.06). When the analysis was limited to the four HWE studies, the association between -1171 5A/6A polymorphism and periodontitis was still significant in the allele model, indicating the robustness of this meta-analysis. Several subgroup analyses were subsequently conducted, and the associations were similar in AgP and CP subgroups in the allele comparison. Additional well-designed studies are required to strengthen our understanding of the association between -1171 5A/6A polymorphism and AgP. Although the minor allele frequency (MAF) and genotype distributions in the included studies for -1171 5A/6A varied by ethnicity (Table 2); the stratified analysis by ethnicity showed a significant association with CP in all three ethnicities. However, all the positive results for the Caucasian and Brazilian mixed populations were derived from a single study. Therefore, future studies with larger samples are warranted to confirm these associations in the Caucasian and Brazilian mixed populations. Tobacco smoking is thought to be an important modifier of the pathogenesis of periodontitis^5^. In further stratified analyses, only the non-smoker subgroup was included to eliminate the possible confounding effects of smoking, and similarly significant findings were found.

The role of *MMP3* in the pathogenesis of periodontitis has been widely investigated. Toyman *et al.* showed that MMP3 levels in the gingival crevicular fluid (GCF) increased in periodontitis cases and played a role in tissue destruction^26^. Recent findings have demonstrated that clinical improvements after non-surgical periodontal therapy are accompanied by a reduction of MMP3 in GCF in both CP and AgP patients^27^,^28^. Some meta-analyses have investigated the associations between *MMP3* -1171 5A/6A polymorphism and systemic diseases^29^,^30^,^31^,^32^,^33^ and have demonstrated that this polymorphism is associated with a decreased risk of gastrointestinal cancer^30^, abdominal aortic aneurysm^31^, myocardial infarction^33^ and head and neck cancer^32^. All of these diseases have been shown to be positively associated with periodontitis in several epidemiological studies^34^,^35^,^36^,^37^. Our results suggest an association between the transcriptionally more active 5A allele and periodontitis, substantiating previous functional findings regarding the *MMP3* rs35068180 variant. We speculate that rs35068180 may influence the development of periodontitis through a similar molecular mechanism. *In vitro* assays of promoter activity have shown that the 5A allele has a two-fold higher promoter activity than the 6A allele^38^. Based on these results, our findings are biologically plausible.

The following limitations of the meta-analysis should be recognised. First, we pooled the data based on unadjusted information, while a more precise analysis needs to be conducted if individual data are available. Second, the number of subjects included in this meta-analysis was relatively small for AgP, Caucasian and Brazilian mixed population subgroups. Hence, the results should be interpreted with caution. Third, the genotypic distribution of the controls also deviated from HWE in three of the studies.

In conclusion, this meta-analysis suggests that *MMP3* -1171 5A/6A polymorphism contributes to a decreased susceptibility to CP, especially in Asian populations. Furthermore, this polymorphism may decrease the risk of AgP in Asians. Replication studies of our results in large independent populations are necessary to validate this association.

## Methods

### Search strategy

Preferred Reporting Items for Systematic Reviews and Meta-Analyses (PRISMA) guidelines were followed^39^. A systematic search of studies that addressed the associations between *MMP3* -1171 5A/6A polymorphism and periodontitis was performed. The literature was retrieved in electronic bio-medical databases including Google Scholar, PubMed, Embase, Medline and China National Knowledge Infrastructure (the last search update was 19 Apr 2015). A combination of the terms “Matrix Metalloproteinases”, “*MMP*”, “periodontal diseases”, “periodontitis”, and “polymorphism” were entered both as text words and as Medical Subject Heading components. No language restriction was placed on the search. Manual searches for references cited in published review and original publications were also performed.

### Inclusion and exclusion criteria

The following two inclusion criteria were used for all retrieved articles: (1) use of a case–control, cohort, nested case–control, or cross-sectional design; and (2) genotype distribution or allele frequency was available from the published data for both cases and controls. The exclusion criteria were as follows: (1) lack of usable allele frequency or genotype distribution data; and (2) lack of control subjects.

### Data extraction

Information was carefully extracted independently from all eligible publications by two investigators. Disagreements were resolved through discussion among the authors to achieve a consensus. The following characteristics were collected for each study: the first author’s family name, publication year, ethnicity (categorised as Caucasian, Asian and mixed), numbers of cases and controls, type of periodontitis (CP or AgP), distribution of genotypes and alleles, smoking status, and genotype identification method.

### Study quality assessment

The quality of each study was evaluated independently by two authors according to the modified STROBE quality score systems^40^. Forty assessment items were used for assessing study quality with scores ranging from 0 to 40. Studies were classified as low- (scores of 0–19), moderate- (scores of 20–29) and high-quality (scores of 30–40). Any disagreement was resolved by discussion among the authors to reach a consensus. The details of the modified STROBE quality score system are listed in Supplementary Table S1.

### Statistical analysis

Odds ratios (ORs), along with 95% confidence intervals (CIs), were used to assess the strength of the association between the *MMP3* gene -1171 5A/6A polymorphism and periodontitis risk. The Z-test was used to determine the statistical significance of the pooled OR. The pooled ORs were performed for the following genetic models: the mutant allele *versus* the wild type, homozygote contrast, heterozygote contrast, dominant model and recessive model. In addition, the HWE in the healthy control populations was tested within each study. We applied the chi-squared method to assess if the genotype distribution in the control group was in HWE. Subgroup analyses were also performed for the HWE in controls, type of periodontitis, ethnicity and smoking status.

Both the fixed-effects model (the Mantel–Haenszel method)^41^ and the random-effects model (the DerSimonian and Laird method)^42^ were used to calculate the pooled ORs. For each genetic comparison, the heterogeneity between studies was assessed using the chi-square based Q-test and was quantified by the *I*^*2*^ statistic. The fixed-effects model was used when heterogeneity was not significant (*P*_heterogeneity_ > 0.10 or *I*^*2*^ < 50%); otherwise, the random-effects model was applied. Sensitivity analysis was performed to assess the stability of the association. Begg’s funnel plot^43^ and Egger’s weighted regression method^44^ were applied to evaluate the underlying publication bias. All statistical analyses were conducted with STATA software, version 11.0 (StataCorp, College Station, TX, USA), using two-sided *p*-values.

## Additional Information

**How to cite this article**: Ding, C. *et al.* Matrix Metalloproteinase-3 -1171 5A/6A Polymorphism (rs35068180) is Associated with Risk of Periodontitis. *Sci. Rep.* **5**, 11667; doi: 10.1038/srep11667 (2015).

## Supplementary Material

Supplementary Information

## Figures and Tables

**Table 1:** Characteristics of eligible studies included in the meta-analysis.

First author	Year	Country	Ethnicity	Form of Disease	Sample size (Case/Control)	Distribution of genotypes and alleles						Smoking status	Genotyping method	Quality scores
						Case			Control					
						5A/5A/5A/6A/6A/6A	5A	6A	5A/5A/5A/6A/6A/6A	5A	6A			
Itagaki	2004	Japan	Asian	CP	205/142	5/58/142	68	342	4/38/100	46	238	Non-smokers	TaqMan	31
Itagaki	2004	Japan	Asian	AgP	37/142*	0/17/20	17	57	4/38/100	46	238	Non-smokers	TaqMan	31
Astolfi	2006	Brazil	Mixed	CP	90/103	19/52/19	90	90	8/70/25	86	120	Non-smokers	PCR-RFLP	29
Kobayashi	2009	Japan	Asian	CP	147/303	—	37	257	—	72	534	S/N	DNA chip & PCR	29
Kobayashi	2009	Japan	Asian	AgP	172/303*	—	58	286	—	72	534	S/N	DNA chip & PCR	29
Loo	2011	China	Asian	CP	280/250	154/115/11	423	137	100/135/15	335	165	Non-smokers	PCR-RFLP	28
Letra	2012	Brazil	Mixed	CP	93/299	—	117	69	—	356	242	S/N	TaqMan	30
Letra	2012	USA	Caucasian	CP	67/202	—	78	56	—	193	211	S/N	TaqMan	30
Li	2012	China	Asian	CP	122/532	75/44/3	194	50	213/283/36	709	355	S/N	PCR-RFLP	27

CP, chronic periodontitis; AgP, aggressive periodontitis; S/N, smokers and nonsmokers.

^*^The control group for AgP used the same subjects as the control group for CP. In the pooled analysis, the CP and AgP cases were combined into one group and the control group was counted only one time.

**Table 2:** Minor allele frequency and genotype distribution in different sources of ethnicities.

Source of Ethnicity	Numbers of comparisons	Minor Allele Frequency	5A/5A/5A/6A/6A/6A
Asian	4	0.53	0.34/0.50/0.16
Caucasian	1	0.52	—
Mixed (Brazilian)	2	0.45	0.08/0.68/0.24

**Table 3:** Meta-analysis of the *MMP3* gene -1171 5A/6A polymorphism (rs35068180) on periodontitis risk.

Variables	N (Case/Control)	6A allele *vs.* 5A allele		5A/6A *vs.* 5A/5A		6A/6A *vs.*5A/5A		5A/6A + 6A/6A *vs.*5A/5A		6A/6A vs. 5A/5A + 5A/6A	
		OR (95% CI)	*P*_*het*_, *I*^*2*^ (%)	OR (95% CI)	*P*_*het*_, *I*^*2*^ (%)	OR (95% CI)	*P*_*het*_, *I*^*2*^ (%)	OR (95% CI)	*P*_*het*_, *I*^*2*^ (%)	OR (95% CI)	*P*_*het*_, *I*^*2*^ (%)
All	7 (1213/1831)	**0.70 (0.62, 0.80)**	0.315, 15.0	**0.50 (0.39, 0.65)**	0.221, 31.9	**0.42 (0.25, 0.69)**	0.265, 24.4	**0.49 (0.38, 0.62)**	0.238, 29.1	0.74 (0.53, 1.02)	0.539, 0.0
HWE	4 (721/946)	**0.80 (0.67, 0.96)**	0.655, 0.0	1.58 (0.40, 6.22)	—	1.30 (0.34, 4.94)	—	1.37 (0.36, 5.20)	—	0.85 (0.54, 1.33)	—
Type of periodontitis											
CP	7 (1004/1831)	**0.72 (0.63, 0.82)**	0.163, 34.8	**0.50 (0.39, 0.64)**	0.345, 9.6	**0.41 (0.25, 0.68)**	0.336, 11.3	**0.48 (0.38, 0.62)**	0.317, 14.9	0.77 (0.56, 1.07)	0.438, 0.0
AgP (Asian)	2 (209/445)	**0.66 (0.48, 0.91)**	0.945, 0.0	4.09 (0.21, 80.21)	—	1.84 (0.10, 35.43)	—	2.44 (0.13, 46.28)	—	0.49 (0.24, 1.04)	—
Ethnicity											
Asian	4 (963/1227)	**0.68 (0.58, 0.80)**	0.149, 43.7	**0.52** (**0.40, 0.68)**	0.198, 38.2	**0.45 (0.25, 0.81)**	0.173, 42.9	**0.51 (0.39, 0.66)**	0.202, 37.4	0.71 (0.49, 1.03)	0.357, 2.9
Asian&CP	4 (754/1227)	**0.73 (0.55, 0.96)**	0.052, 61.2	**0.52 (0.40, 0.68)**	0.333, 9.0	**0.44 (0.24, 0.79)**	0.226, 32.8	**0.50 (0.39, 0.65)**	0.284, 20.6	0.75 (0.52, 1.10)	0.260, 25.7
Caucasian (CP)	1 (67/202)	**0.66** (**0.44, 0.97)**	—	—	—	—	—		—		—
Brazilian Mixed (CP)	2 (183/402)	0.80 (0.62, 1.04)	0.477, 0.0	**0.31 (0.13, 0.77)**	—	**0.32 (0.12, 0.89)**	—	**0.31 (0.13, 0.76)**	—	0.83 (0.42, 1.64)	—
Smoke											
Non-smokers	3 (612/495)	**0.73 (0.60, 0.88)**	0.414, 0.0	**0.54 (0.39, 0.75)**	0.151, 47.1	**0.50 (0.28, 0.89)**	0.260, 25.8	**0.53 (0.39, 0.73)**	0.189, 40.0	0.80 (0.57, 1.13)	0.825, 0.0

N, Number of comparisons; OR, odds ratio; 95% CI, 95% confidence interval; CP, chronic periodontitis; AgP, aggressive periodontitis.
