# Supplementary material for: Matrix Metalloproteinase-3 -1171 5A/6A Polymorphism (rs35068180) is Associated with Risk of Periodontitis
Source: Sci Rep. 2015 Jun 30;5:11667. doi: 10.1038/srep11667 (PMC4485030; doi:10.1038/srep11667)
Supplement: Supplementary Information [file srep11667-s1.pdf]

Matrix Metalloproteinase-3 -1171 5A/6A Polymorphism (rs35068180) is Associated with Risk of Periodontitis

Cheng Ding[1], Xing Chen[1], Peng-tao Zhang[1], Jin-ping Huang[2], Yan Xu[3], Ning Chen[4,*] & Liang-jun Zhong[1,5,*]

Criteria items	Score (0 to 40)
Title and Abstract	☐ 0 ☐ 1
Title and Abstract: Indicate the study design (case-control or cohort study) in the title or the abstract	☐ 0 ☐ 1
Abstract: Provide an informative and balanced summary of the study	☐ 0 ☐ 1
Introduction: Explain the scientific background and rationale for the investigation	☐ 0 ☐ 1
Introduction: State specific objectives, including any prespecified hypotheses	☐ 0 ☐ 1
Methods	☐ 0 ☐ 1
Study Design: Present key elements of study design	☐ 0 ☐ 1
Setting: Describe the setting, locations, and relevant dates, including periods of recruitment, exposure, follow-up, and data collection	☐ 0 ☐ 1
Participants: Give the eligibility criteria of case	☐ 0 ☐ 1
Participants: Give the sources and methods of case ascertainment and control selection	☐ 0 ☐ 1
Participants: Give matching criteria and the number of controls	☐ 0 ☐ 1
Variables: Clearly define all outcomes, exposures, predictors, potential confounders, effect modifiers	☐ 0 ☐ 1
Data sources/Measurement: Give sources of data and details of methods of assessment	☐ 0 ☐ 1
Data sources/Measurement: Describe comparability of assessment methods	☐ 0 ☐ 1
Bias: Describe any efforts to address potential sources of bias	☐ 0 ☐ 1
Study size: Explain and describe the estimation of the study size	☐ 0 ☐ 1
Quantitative variables: Explain how quantitative variables were handled in the analyses	☐ 0 ☐ 1
Quantitative variables: Give group included criteria in the analyses	☐ 0 ☐ 1
Statistical methods: Describe all statistical methods, including those used to control for confounding	☐ 0 ☐ 1
Statistical methods: Describe any methods used to examine subgroups and interactions	☐ 0 ☐ 1
Statistical methods: Explain how missing data were addressed	☐ 0 ☐ 1
Statistical methods: Explain how matching of cases and controls was addressed	☐ 0 ☐ 1
Statistical methods: Describe any sensitivity analyses	☐ 0 ☐ 1
Hardy-Weinberg equilibrium: HWE was assessed	☐ 0 ☐ 1
Hardy-Weinberg equilibrium: HWE of control group was assessed	☐ 0 ☐ 1
Results	
Participants: Report the numbers of individuals at each stage of the study, such as numbers potentially eligible, examined for eligibility, confirmed eligible, included in the study, completing follow-up and analyzed	☐ 0 ☐ 1
Participants: Give reasons for non-participation at each stage	☐ 0 ☐ 1
Participants: Give a flow diagram	☐ 0 ☐ 1
Descriptive data: Give characteristics of study participants (e.g. demographic, clinical diagnosis, ethnicity, sex ratio, etc.)	☐ 0 ☐ 1
Descriptive data: Indicate the number of participants with missing data	☐ 0 ☐ 1
Outcome data: Report numbers in each exposure category, or summary measures of exposure	☐ 0 ☐ 1
Main results: Give unadjusted estimates and confounder-adjusted estimates and their 95%	☐ 0 ☐ 1

confidence intervals		
Main results: Make clear which confounders were adjusted for and why they were included	☐ 0	☐ 1
Main results: If relevant, consider translating estimates of relative risk into absolute risk for a meaningful time period	☐ 0	☐ 1
Other analyses: Report other analyses such subgroups, interactions, and sensitivity analyses	☐ 0	☐ 1
Discussion	☐ 0	☐ 1
Key results: Summarize key results with reference to study objectives	☐ 0	☐ 1
Main results: Report category boundaries when continuous variables were categorized	☐ 0	☐ 1
Limitations: Discuss both direction and magnitude of any potential bias	☐ 0	☐ 1
Interpretation: Give a cautious overall interpretation of results considering objectives, limitations, multiplicity of analyses, results from similar studies, and other relevant evidence	☐ 0	☐ 1
Generalizability: Discuss the generalizability (external validity) of the study results	☐ 0	☐ 1
Other	☐ 0	☐ 1
Funding: Give the source of funding and the role of the funders for the present study	☐ 0	☐ 1

Supplementary Table S1. The modified STROBE quality score systems.
